# Integration of a personalised mobile health (mHealth) application into the care of patients with brain tumours: proof-of-concept study (IDEAL stage 1)

**DOI:** 10.1136/bmjsit-2021-000130

**Published:** 2022-12-22

**Authors:** Andrew Gvozdanovic, Felix Jozsa, Naomi Fersht, Patrick James Grover, Georgina Kirby, Neil Kitchen, Riccardo Mangiapelo, Andrew McEvoy, Anna Miserocchi, Rayna Patel, Lewis Thorne, Norman Williams, Michael Kosmin, Hani J Marcus

**Affiliations:** 1 Victor Horsley Department of Neurosurgery, National Hospital for Neurology and Neurosurgery, London, UK; 2 Department of Oncology, University College London Hospitals NHS Foundation Trust, London, UK; 3 Vine Health, London, UK; 4 University College London Division of Surgery and Interventional Science, London, UK

**Keywords:** health technology, patient outcome assessment, process assessment (health care), health care quality, access, and evaluation

## Abstract

**Objectives:**

Brain tumours lead to significant morbidity including a neurocognitive, physical and psychological burden of disease. The extent to which they impact the multiple domains of health is difficult to capture leading to a significant degree of unmet needs. Mobile health tools such as Vinehealth have the potential to identify and address these needs through real-world data generation and delivery of personalised educational material and therapies. We aimed to establish the feasibility of Vinehealth integration into brain tumour care, its ability to collect real-world and (electronic) patient-recorded outcome (ePRO) data, and subjective improvement in care.

**Design:**

A mixed-methodology IDEAL stage 1 study.

**Setting:**

A single tertiary care centre.

**Participants:**

Six patients consented and four downloaded and engaged with the mHealth application throughout the 12 weeks of the study.

**Main outcome measures:**

Over a 12-week period, we collected real-world and ePRO data via Vinehealth. We assessed qualitative feedback from mixed-methodology surveys and semistructured interviews at recruitment and after 2 weeks.

**Results:**

565 data points were captured including, but not limited to: symptoms, activity, well-being and medication. EORTC QLQ-BN20 and EQ-5D-5L completion rates (54% and 46%) were impacted by technical issues; 100% completion rates were seen when ePROs were received. More brain cancer tumour-specific content was requested. All participants recommended the application and felt it improved care.

**Conclusions:**

Our findings indicate value in an application to holistically support patients living with brain cancer tumours and established the feasibility and safety of further studies to more rigorously assess this.

**WHAT IS ALREADY KNOWN ABOUT THIS TOPIC**

- mHealth applications are increasingly used both with and without healthcare provider supervision. Although they have potential to positively impact patient care, most applications fail to validated with clinical evidence.

**WHAT THIS STUDY ADDS**

- A first of its kind in evaluating the clinical benefits of mHealth applications in the neurosurgical care of patients with brain cancer. It describes the stepwise approach to mHealth evaluation using the IDEAL framework.

**HOW THIS STUDY MIGHT AFFECT RESEARCH, PRACTICE OR POLICY**

- This study will act as a guide to validation and evaluation of mHealth applications using the IDEAL framework.

## Introduction

Brain cancers make up 3% of cancers worldwide but have a disproportionate impact on the healthcare systems that treat them; patients have higher rates of mortality and their cancers are inherently disabling.^1–3^ In view of the significant morbidity despite best management, there is a need to fully optimise quality of life; both in patients undergoing curative treatment and palliative care.^4^

Health-related quality of life (HRQoL) is a multidimensional concept encompassing the physiological, psychological and social components of well-being.^5^ Validated methods aimed at capturing HRQoL such as (electronic) patient-recorded outcome measures ((e) =PROMs) have been designed for general health (EuroQol 5 dimensions of health questionnaire; EQ-5D-5L and the Short Form 36 Health Survey; 3SF-36) but also for specific disease states (European Organisation for Research and Treatment of Cancer Brain Tumor Questionnaire; EORTC-QLQ BN20 and FACT-Br for brain cancer).^6–8^ Since 2009, the integration of PROMs into patient care has been driven by the UK Department of Health via its PROMs programme; this initiative recommends all patients have routine measurements taken before and after receiving surgery, although this is not mandated in all procedures at present.^9^ The development of web and smartphone-based platforms has enabled these to be distributed remotely; for example, a feasibility study by Laccetti *et al* used a mobile based remote patient monitoring platform to enable longitudinal predictive modelling in prostate cancer.^10^ However, a number of studies have demonstrated that although digital technologies may improve accessibility, it brings with it a number of barriers both in the lack of IT infrastructures to support their distribution and collection, but also in their difficulties for patients to use and complete.^11–13^

One of the biggest challenges in supporting the patient experience out of hospital has classically been communication and data sharing.^14^ The English National Institute for Health and Care Excellence provides guidance specifically regarding the organisation of healthcare services for people with a brain tumour diagnosis highlighting the importance of (1) high-quality written information in patient care and (2) considering web-based information systems to increase data access and sharing among healthcare providers (HCPs).^15^ One of the most promising modalities to help facilitate this is the integration of mHealth technologies.

mHealth technologies are a promising platform by which we can support the multidimensional needs of patients living with brain cancer. Tools such as smartphone applications provide a convenient and accessible way for patients to both record and receive healthcare data. In the oncology setting, mHealth platforms have been proposed to monitor symptoms, treatment side effects and their severities.^16 17^ These applications may also be used to distribute personalised patient centric educational material.^16 18^ mHealth improves on traditional symptom diaries by tracking patients in real-time facilitating earlier deterioration detection and intervention.^19^ Thus, mHealth applications have the potential to not only improve clinical outcomes, but may potentially lower operational costs by reducing morbidity, admissions and length of inpatient stays.^20^

A total of 47 000 mHealth applications were listed on the Google Play Store in 2020, but it is unclear how many of these have been evaluated for efficacy, safety or effectiveness. For example, a review of medication adherence applications looked at 5881 applications available, finding only 5 having any sort of evidence base for their effectiveness.^21^ Some of these applications may need to be regulated as software as a medical device under the Medicines and Healthcare products Regulatory Agency (MHRA) in the UK and the Food and Drug Associate in the USA. However, many will fall outside of this remit by branding themselves as ‘wellness’ tools and thus do not have to conform to stringent review.^22^ As mHealth uptake is relatively new, and gold standard evaluation (Randomised Control Trials; RCTss) can be difficult and expensive to run, there is a lack of robust evidence behind these tools—although frameworks are becoming increasingly common.^22–24^

Where studies have been carried out, the results are promising. For example, a multicentre randomised trial carried out by Denis *et al* in patients with lung cancer demonstrated symptom monitoring via weekly web-based ePROMs following treatment was associated with increased survival compared with standard imaging surveillance.^25^

Vinehealth is an mHealth application supporting patients to learn more about their disease and contribute to their clinical care and decision-making. The platform aims to deliver highly personalised behavioural interventions through machine learning and evidence-based educational material to support self-management. Vinehealth facilitates communication with clinical teams by enabling patients to track symptoms, side effects, medications, appointments and lifestyle. Patients are also able to complete validated PROMs. Data can be collected actively, or passively through integration with smart devices such as watches to collect physical activity. Vinehealth was chosen for this study as it attempts to addresses the multimodal (eg, symptom tracking, medication tracking, or physical activity monitoring), needs of patients living with brain cancer.

We; therefore, designed a proof-of-concept study that aimed to:

1. Assess the feasibility of a larger study evaluating the impact of the Vinehealth application in brain tumour care.
2. Assess engagement and thus, the applications ability to: (1) collect real-world and validated ePROM data (2) deliver educational content.
3. Assess subjective opinion on improvement in care.
4. Generate user feedback to improve intervention and inform future study design.

The results of this study will provide the basis to inform the development and design of larger, more robust studies assessing the impact of the Vinehealth application.

## Methods

### Design

A mixed-methodology design was adopted as defined by the IDEAL guidelines for assessing innovation in surgery. In line with the framework for an IDEAL stage 1 study, single digit participant numbers were planned for the study, in order to evaluate safely if the methods trialled are of value for further study, and the relevant reporting guidelines used in the preparation of this manuscript.^26^ Patients from a single site undergoing surgery for brain cancer were approached to participate until 6 were recruited. Following recruitment, patients were onboarded to the app (invited to download and sign up to the application). Onboarded patients were followed up over a 2-week period for the collection of patient feedback. After this, patients were asked to give further feedback (informal discussion led by the participant, each lasting 45 min to an hour) over the phone (postdischarge) at which point formal contact ceased (offboarded). Data collection then continued via the Vinehealth application (12 weeks total data collection from onboarding) to assess for ongoing engagement.

### Participants and setting

The inclusion criteria were as follows: (1) aged 18 or over, (2) had a proficient English speaking and reading ability, (3) owned a smartphone running either Android V.4.4+ or iOS V.9.2+, (4) had a prospective brain cancer diagnosis and (5) were booked for or had undergone neurosurgical management within 1 week of recruitment and orientation with the app (onboarding).

All patients were booked or had undergone surgery by surgeons who have a subspecialty interest in neuro-oncology. All operations took place at the National Hospital for Neurology and Neurosurgery, which is a large regional neuroscience centre, that carries out approximately 150–200 brain cancer operations each year.

Patients were recruited over a 2-week period from October 2020 to November 2020, when the UK remained in the COVID-19 pandemic. Recruitment was approached pragmatically, with daily departmental check ins for new urgent referrals/transfers and patients to come in.

### Intervention

**Figure 1:**
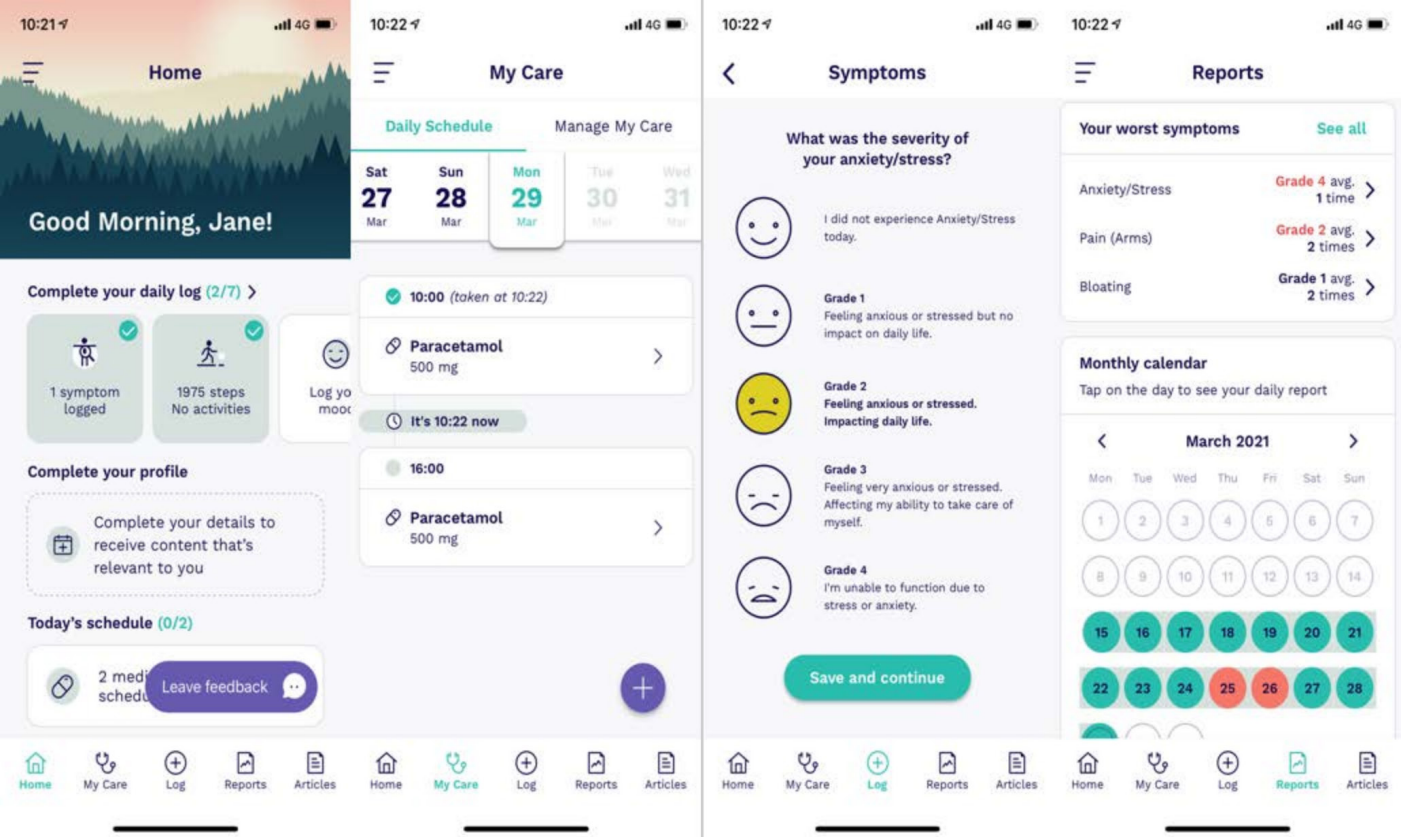
Images demonstrate the Vinehealth application including the home screen where different activities, digital therapeutics and information can be quickly assessed. The My Care page allows patients to view their medications and appointments on a specific day. A visual representation of symptom logging with a Visual Analogue Scale is visible. The report screen displays an overall summary of all of the data a user has recorded in a time period, accessible via a calendar view.

The Vinehealth Application is a smartphone application using behavioural science and machine learning driven personalisation to improve the self-management and psychological well-being of people living with cancer. Within the study, patients were asked to interact with the entire application as much as possible, with a view to determining its usefulness. A collection of screenshots displaying the application can be seen in figure 1.

The ‘Daily Log’ consists of the following:

1. ‘How are you’—A Visual Analogue Sliding Scale (VASS) ranging from 0% to 100%, with 0% corresponding to ‘I am struggling’ and 100% corresponding to ‘I feel fully in control’.
2. Symptom log—A predefined collection of symptoms based on the ‘Common Terminology Criteria for Adverse Events (CTCAE), which itself has a VASS for symptom severity on a 4-point scale based on CTCAE and 0–100% severity.
3. Activity log—A module which can automatically pull steps from the native functioning of the health tool of the phone and can be enhanced with a self-input of a predefined activity, with duration and ‘notes’.
4. Temperature log—Captures recorded room temperature and time, with notes, optional input.
5. Weight log—Captures recorded weight through a third-party device with time taken.

The ‘My Care’ module consists of the following:

1. Medication tracker—Including medication name, dose, units of dose, frequency±time of day to be taken, and notes.
2. Appointment tracker—Including appointment type, date, time, location and ‘with whom’, as well as notes.

The ‘Articles’ section consists of a collection of cancer agnostic educational material as well as content aimed more to their specific cancer needs.

### Outcomes

#### Primary outcome

(1) Feasibility of a larger study—A pragmatic approach was been taken to assess the feasibility of a larger study evaluating the Vinehealth application. Factors, such as recruitment, retention rate, and number of pathway and application modifications, were taken into consideration.

#### Secondary outcomes

(1) Engagement with data collection—The total number of real-world and ePROM data points collected throughout the study was tallied and percentage change over time calculated.

Vinehealth real-world data collection: Patients were able to self-record data in domains such as symptoms, medications, physical activity and well-being via the application. The data were not monitored in real time. As such, the application acted as a digital diary visible to the patient throughout the study with the knowledge that this would be analysed (deidentified) at the end of the study period.

ePROs: All participants were asked to complete three validated (e)PROMs: EORTC-BN20 (European Organisation for Research and Treatment of Cancer Brain Tumor Questionnaire), EQ5D5L (EuroQol 5 dimensions of health questionnaire) and PAM (Patient Activation Measure). These were expected to be completed around day 0±3 of onboarding and then again 2 weeks later ±3 days. The PAM was completed on paper while the EORTC-BN20 and EQ5D5L were integrated into the Vinehealth application (the digital versions of these PROMs has gone through a validation process and approved by their respective bodies).

(2) Subjective improvement in care—This was assessed using both semistructured interviews before and after application use, as well as through mixed-methodology surveys, again performed at onboarding and offboarding.

Semistructured interviews: All participants were to be included in an onboarding and offboarding semistructured interview, which took place at day 0±3 from onboarding and day 14±3 from offboarding. Interviews were carried out in person and over the phone.

Mixed-methodology surveys: All participating members were asked to complete an onboarding and offboarding survey. Where required, a Likert scale ranging from 1 to 10 was implemented with an explanation (ie, 1 is strongly disagree and 10 is strongly agree) applied to each statement or question.

(*3)* User feedback—Semistructured interviews were used to establish general feedback about the application and areas for improvement in future studies. This was also captured in more depth via onboarding and offboarding questionnaires, breaking down each section for evaluation.

Semistructured interviews: This interview aimed to explore in more detail (1) problems they faced holistically in their health at that time, (2) potential ways that a mobile health tool could support them and their health problems, and for offboarding and (3) their experience of the Vinehealth application and its impact.

Mixed-methodology surveys: The onboarding survey was broken down into four parts: (1) demographics, (2) familiarity with healthcare services, (3) familiarity with health technology and (4) expectations.

Expectations were broken down into (1) symptom tracking, (2) medication tracking, (3) appointment tracking, (4) impact on day-to-day life, (5) educational content and (6) engagement with care. Each section contained a number of statements related to how they expected to use components of the Vinehealth application.

The offboarding survey captured whether the participant had (1) used a specific module, (2) whether they had found it useful and (3) the degree to which it helped them to better understand their health/improved their care. Again, this was achieved via a 10-point Likert scale. For each section, qualitative feedback (limited to 3 positive and 3 negative points) was captured.

### Data analysis

Following an initial screen, responses were parsed for themes. Each completed interview was assigned an identifying label based on chronological order of completion, that is, ‘participant 1’, ‘participant 2’, etc.

Patient onboarding and offboarding questionnaire scores were reviewed in tandem with the offboarding semistructured interview. Again, each completed interview and questionnaire was identified and labelled for concurrent comparison. A descriptive thematic analysis was carried out based on surveyed response and verbal feedback to identify core themes.

PAM scores were collected via the paper Patient Activation Measure PAM 13 British. Results were generated by sending the anonymised data to the Insignia Health server (Cardiff, Wales), no patient identifiable data were sent to the servers and only the anonymised PAM data is stored. In cases where a PAM response has more than 3 N/A answers, it is considered unreliable and is given a default score of 51 and PAM level 2.

EQ5D5L and EORTC-BNO were calculated as per their respective guidance and provided algorithms. For the EORTC QLQ-BN20, all of the scales and single-item measures range in score from 0 to 100. A high score for the scales and single items represents a high level of symptomatology or problems.

## Results

### Recruitment

Six patients who agreed to take part in the study were the first to be approached; no patients declined to be involved. Four were female and two were male, all were aged between the ages of 45 and 69. All participants were planned for or had recently undergone (operation±3 days) surgical management of their brain cancer.

Of those that completed the onboarding questionnaire, three of five had previous experience with medical treatment of a chronic or long-term health problem. Of these, all had previous experience with the National Health Service (NHS), and one had previously been treated under the private system as well. On a Likert scale where 1 is poorly and 10 is highly, all participants ranked their previous healthcare experience 8 or above.

All participants who completed the questionnaire felt confident in using mobile phone applications in their everyday life and all had previously used mobile health applications. mHealth applications in use included: (1) NHS COVID-19, (2) NHS app, (3) Fitbit: Health & Fitness, (4) MyCare-UCLH and (5) Patient access. Three of the five participants who completed the onboarding questionnaire owned a smart health device at the start of the study these included an Apple Watch, a Fitbit and a Samsung Galaxy smartwatch. One participant bought a smartwatch specifically for use in the study.

### Feasibility

No overt problems arose in recruitment, however, time constraints meant that not all questionnaires/surveys/interviews could be completed if a patient was admitted on the day of procedure.

In total, four of six patients were retained throughout the study period (66.6% retention rate) and no participants who downloaded the Vinehealth application dropped out. Following consent, one patient withdrew from the study postoperatively without filling in any questionnaires, PROMs or semistructured interview. This participant stated they felt unable to commit to the demands of a study during such an overwhelming period in their life. One participant withdrew from the study postoperatively completing only the onboarding questionnaire and PAM; no reason was provided.

No intrastudy adjustments were carried out. No risks, harms or safety concerns were identified. Prior to onboarding, participants were informed that the application was not monitored and it was akin to a diary; it was reiterated that it did not replace normal treatment from the medical team. They were advised to raise any clinical concerns (as normal) with the treating medical team directly.

### Clinical characteristics

Four participants remained in the study, three female and one male, aged between 45 and 69 years. All participants were fully independent, with a performance status of 0, prior to admission for surgery and further treatment. All four underwent craniotomy and resection of tumour, with or without adjuvant chemoradiotherapy (table 1). The diagnoses of the four participants were glioblastoma (n=2), metastasis from triple-negative breast carcinoma (n=1) and haemangioblastoma (n=1).

**Table 1:** Clinical characteristics of participants enrolled in study

Participant	Performance status	Treatment received before study enrolment	Neurosurgical treatment during study	Final diagnosis	Adjuvant treatment
1	0	Lumpectomy and chemoradiotherapy; Posterior fossa craniotomy and resection left cerebellar metastasis	Craniotomy and resection of right frontal metastasis	Left frontal metastasis from primary breast carcinoma (triple negative)	Chemoradiotherapy
2	0	Nil	Posterior fossa and resection of right cerebellar lesion	Right cerebellar WHO grade 1 haemangioblastoma	Nil
3	0	Nil	Craniotomy and resection of left parietal tumour	Left parietal glioblastoma	Chemoradiotherapy
4	0	Nil	Craniotomy and resection of right frontal tumour	Right frontal glioblastoma	Radiotherapy (chemotherapy stopped at induction due to renal function)
5	0	Nil	Craniotomy and biopsy of left temporal lesion	Left temporal glioblastoma	Chemoradiotherapy
6	1	Pneumonectomy and chemoradiotherapy	Craniotomy and resection of left frontal lesion	Left frontal metastasis from primary lung squamous cell carcinoma	Whole brain radiotherapy

### Engagement

Of the four participants retained throughout the study period, one completed all tasks within the predetermined timeframe. Due to poor health, one patient’s offboarding was not completed within the predetermined time frame. A summary of completion can be found in table 2.

**Table 2:** Summary of tasks completed by participants enrolled in study

Patient	Onboarding					Offboarding				
	Ques.	SSI	PAM	EQ-5D	BN20	Ques.	SSI	PAM	EQ5D	BN20
1	X	X	X	X	X	X	X	X	X	–
2	X	X	X	–	–	X	X	X	X	X
3	X	W	X	W	W	W	W	W	W	W
4	W	W	W	W	W	W	W	W	W	W
5	X	X	X	–	–	/	/	/	–	–
**6**	**X**	**X**	**X**	**X**	**X**	**X**	**X**	**X**	**X**	**X**

Overall, there was a 79% retained completion rate for questionnaires and 71% retained completion rate for semistructured interviews within the predetermined timeframe.

/, completed within 1 week of intended time frame; –, not completed or completed outside intended time frame; EQ-5D-5L, EuroQol 5 dimensions of health; PAM, patient activation measure; Ques, questionnaire; SSI, semistructured interview; W, withdrew; X, completed within intended time frame.

**Figure 2:**
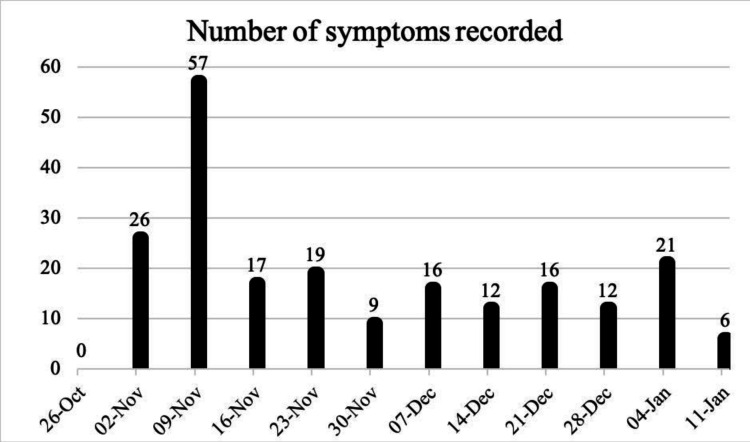
Total number of symptoms recorded in a 7-day (week) period over 12 weeks.

Prior to application use, all participants who completed the questionnaires stated they would use a mobile application to record their symptoms and side effects. In total, 211 symptoms and side effects were logged with a peak of 57 and a low of 6 symptoms logged as demonstrated in figure 2.

Prior to application use, all participants felt they would use a mobile application to help record the frequency by which they took their medication. All participants taking part in the offboarding questionnaire stated they had regular medications to take during the study. One of the four participants used the medication tracker in the study period and recorded 47 doses out of a potential 342 (12.1%).

All participants had appointments during the study period, but none used the appointment tracking feature.

**Figure 3:**
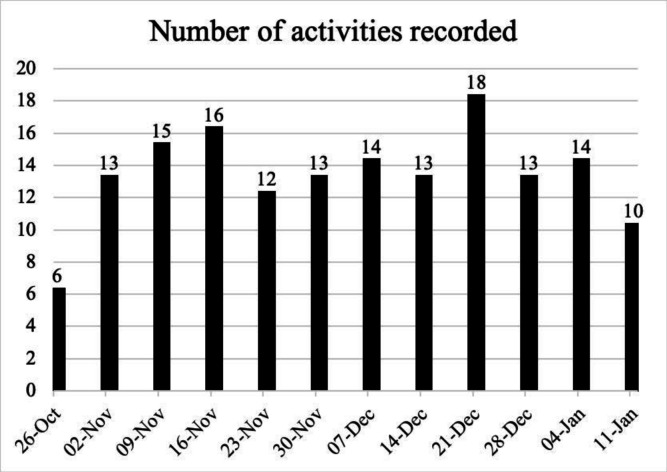
Demonstrates the total recorded activities (including steps) in a 7-day (week) period over 12 weeks.

A total of 157 activities were recorded over the course of the 12-week study period seen in figure 3. The total interactions recorded averaged 13 (mean), with a peak of 35 064 steps and a low of 0 steps recorded in a week.

All participants felt they would use a mobile application to answer questions about how their cancer was impacting their health. The EQ5D5L and EORTC-BN20 were both presented via the Vinehealth application and had a 42% (54% retained) and 33% (42% retained) completion rate, respectively. Response was impacted by a technical issue which affected delivery of the questionnaires—this has since been fixed. All those who received EQ5D5L and EORTC-BN20 had a completion rate of 100%. All participants stated they would use a mobile application to answer questions about their engagement with treatment and thought this would be helpful to them and their healthcare team. The PAM questionnaire had a completion rate of 67% (79% retained). All questionnaires and surveys started were completed in full.

**Figure 4:**
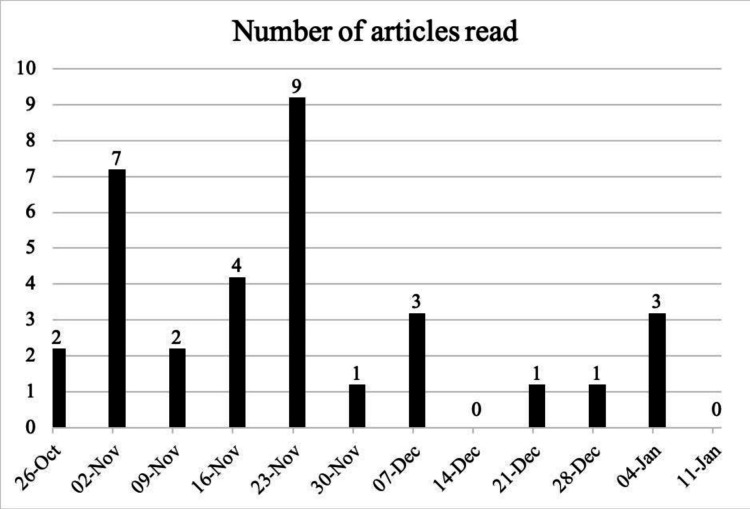
Total number of articles read in a 7-day (week) period over 12 weeks.

All participants surveyed stated they would like a mobile health application to provide them with relevant content about their diagnosis, treatment and lifestyle. All offboarded patients except one used the educational content. A total of 33 articles were read throughout the study with a peak of 9 and a low of 0 read in a week. An average of 2.75 articles were read in total a week. Use of educational articles is demonstrated in figure 4.

**Figure 5:**
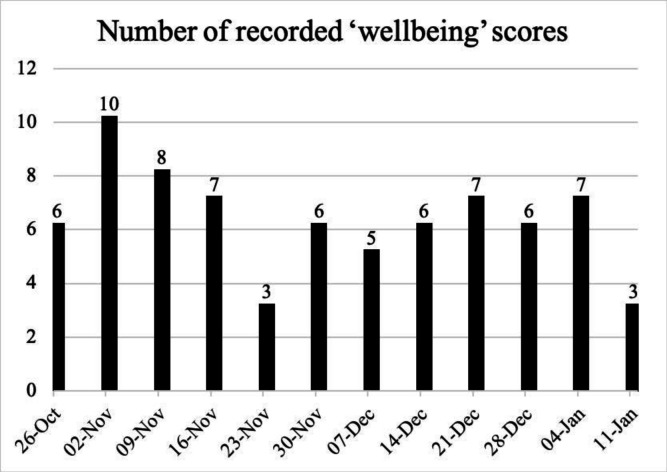
Total number of ‘well beings’ recorded in a 7-day (week) period over 12 weeks.

Seventy-four well-being scores were submitted throughout the study period with a peak of 10 and a low of 3 in a week demonstrated in figure 5. Well-being scores averages 59.75% in week 1 increasing to 84% in the final week of the study where 0% is the lowest well-being and 100% is the highest subjective well-being.

### Subjective improvement in care

Prior to downloading the Vinehealth application, all participants completing the onboarding questionnaire thought that symptom tracking would help them and their HCP to better understand their health. All application users logged symptoms within the Vinehealth application, found this useful, and thought it helped them to better understand their health. Participants appreciated the visual aspect of being able to view trends in their symptoms, the ability to scale their symptom severity (also visually).

Prior to application use, 80% of participants felt confident in remembering to take their medication. All surveyed participants felt that the idea of a medication tracker was a good idea and particularly liked the idea of an alert to remind them to take their medications. All participants felt that capturing their adherence would be beneficial both to themselves and their HCP. However, only one participant used the medication tracker and did not feel it had impacted their care as it had not been used reliably.

Prior to application use, all participants surveyed felt the Vinehealth application would be a helpful way to set reminders about their medical appointments. Eighty per cent participants felt confident in remembering their medical appointments without an application. During the study, none of the four application users used the appointment tracking.

Two participants surveyed prior to application use thought answering questions about disease impact on their daily life would help them and their HCPs to better understand their health. One participant though it would be neither useful or non-useful and one thought it would be non-useful. Two participants felt that recording how their diagnosis was impacting their everyday life would help them to better understand their health, one though this would be neither useful or non-useful and one thought it would not be useful. Average EQ-5D-5L (EuroQol 5 dimensions of health) score at the start of the study was 2.07±1.28 (average±SD) and 1.73±1.22 at the end of the study. Average QLQ-BN20 (Quality of Life Questionnaire in Brain Cancer) score at the start of the study was 13.33 and 22.5 at the end of the study.

Three participants thought it was useful to measure their engagement with PAM while one thought it was neither useful nor non-useful. All participants thought the Vinehealth application made them more engaged with their care and empowered them to make decisions about their health.

Onboarding PAM scores ranged from 33 to 67.8 and levels of 1–3. Offboarding PAM scores ranged from 42.2 to 75 and levels of 1–3 remained static. The four participants who completed both onboarding and offboarding questionnaires saw a change in PAM score by 1.57, 0.86, 1.12 and 1.28 (1.21 mean average).

Two of the three who used the educational content found it useful, the third felt it was neither useful nor non-useful. Two participants thought the educational content was largely not specifically relevant to brain cancer while one thought it was relevant.

At the end of the study, all participants stated that they felt that using the VineHealth app had improved their care.

### Interview: user feedback

After analysing participant responses and parsing for themes, six main topics emerged from discussions: (1) nutrition, (2) activity, (3) symptoms and mood, (4) medications, (5) educational content and (6) general impressions.

#### Nutrition and lifestyle

All participants raised nutrition as an important component of their holistic health needs. This ranged from using the Vinehealth application to look for ways to improve their diet via the educational content, but also in monitoring their weight, which was affected by corticosteroids. Three out of four participants used the Vinehealth application in order to subjectively improve their general lifestyle.

‘I really enjoyed the sleeping and breathing sections, it was easy to use and now I do things like turn off notifications, and put my phone on do not disturb which has helped me sleep’.

#### Vitals and activity

Three out of four users used a wearable device with their Vinehealth application; one participant bought a smart device to capture their vitals and activity. Participants expressed that they liked the direct integration with both their google fit and apple health applications. Participants expressed that they monitored aspects of their vital signs such as heart rate and blood pressure regularly. Activity was something all participants were focused on due to fatigue in the perioperative period.

‘Because of my initial lack of activity, it was really encouraging to see the visual improvement of not going out to becoming more active again.’

#### Symptoms and mood

Three out of four participants described low mood in the perioperative period ranging from anxiety and stress to low mood and depression. All participants expressed that the symptom tracking was one of the best features of the application, although one patient felt that it failed to stand out among other alternative applications. Overall, no improvements to this module were felt to be required. A lack of feedback from HCPs to the symptoms tracked was noted and none of the participants actively shared their application data with their HCPs.

‘I’ve had lots of occupational health and mental health input, I’ve used this app [Vinehealth] like a diary; it’s comforting to have a support tool which is easily available and so visual.’

#### Medications

All participants expressed that a medication tracker would be useful, but two of four felt that they would not use it at present. Two participants expressed wanting to try the Vinehealth medication tracker, but one stated they were put off by having to input all their medication themselves. The one participant who had attempted to use the medication tracker said the process seemed too complex. Feedback generally revolved around a desire for an automated process by which medications would be added to the application.

‘I didn’t use the medication tracker because there were just too many medications I would have had to put on—it would take too long to use’.

#### Educational content

Participants liked the perioperative information and its inclusion of holistic learning content such as diet and lifestyle. It was felt that the educational content available was less relevant to brain cancer care and its treatment.

‘Some of the content was really useful and helped me think about my diet but some just didn’t seem relevant’.

#### General feedback

Of the four users asked, all would recommend the Vinehealth application and felt mobile applications would be able to help improve patient cancer care. All four users appreciated the visual nature of the application but thought content could be better directed to brain cancer care. Of the two participants that withdrew from the study, one felt that in retrospect, it was not an appropriate time to join a study; the other did not provide feedback as to why they withdrew.

‘It’s a very useful tool for me, absolutely a 9/10’.

## Discussion

### Principal findings

There is enormous potential for the use of mHealth applications to collect and distribute data, to empower patients and support clinical decision-making. However, there is a serious lack of published evidence to support any of these claims, particularly regarding improved clinical outcomes.

To the best of our knowledge, this is the first prospective study assessing the feasibility of integrating a mobile smartphone intervention into the care of patients living with brain tumours. Although a number of web-based and mobile health applications are available, the Vinehealth application is one of the few to provide support in all aspects of a patient’s biopsychosocial health.

Over a 12-week period, the Vinehealth application was able to collect real-world data while supporting the patient experience. The size of this study means we cannot extrapolate the true impact and potential value of this tool, but it provides the pivotal first step in ensuring the safe and robust evaluation of a new clinical care adjunct in the neurosurgical cancer care pathway.

mHealth technology has the potential for a broad range of adverse events. However, no safety concerns were raised with the use of this application. Though not designed as an efficacy study, we were able to demonstrate a 100% subjective improvement in care in those using Vinehealth.

Older adults with chronic health conditions have been shown to benefit from remote patient monitoring, however, use of technology has shown to be challenging.^14^ The incidence of brain and CNS cancers is highest in those aged over 40 years with a median age at diagnosis of 59 years.^27^ In a single centre, we were able to recruit roughly 3 patients a week with a retention rate of ~66%. All of those recruited were over 40 (range 45–69 years) and had some mobile health familiarity either through smartphone applications or smart devices such as watches. Thus, in our recruited cohort, we demonstrated that the integration of a digital health application was not a particularly novel or alien a concept. Generally, there were few usability issues indicating that the application is geared appropriately to the user experience of the cohort. Although there has been a steady increase in the uptake of digital health technologies in older patients, functional impairment can impact access and thus, exclude the patients who would value it most.^28 29^ To demonstrate appropriate usability and acceptance more robustly, further assessment is required with a larger more expansive cohort to reflect our target demographic more accurately.

### Comparison to other studies

A few studies are directly comparable for review; most applications focus on a single aspect of care (data tracking or digital therapeutics) or different disease cohorts (cardiovascular health and diabetes). A comparison that can be made is retention and usage rates. In our study, 66% of recruited patients adopted the intervention with a 75% retention in app use following cessation of formal follow-up (postoffboarding survey); 50% of users continued to use the application at 11 weeks. This is broadly similar to a number of other studies looking at mobile application integration: in breast cancer (69% adoption and 73% retention), endometrial and breast cancer (75%–77% retention), oesophageal (80% usage) and colon cancer (73% usage).^30–33^ Of note, two studies within the neurosurgical space of traumatic brain injury and smartphone applications, have chosen to also assess usability, something that was outside the scope of this feasibility evaluation.^34 35^ The inclusion of usability may be something to consider in future studies. Regarding retention and usage again, the paper by Juengst *et al* assessing feasibility of an mHealth system in traumatic brain injury (TBI) had similar completion rate of ePROs (Patient Health Questionnaire-9; PHQ-9 and General Anxiety Disorder-7 questionnaire; GAD-7) at 73.4%.

### Strengths and limitations

By carrying out a small but focused study, we have been able to gain in depth feedback on the Vinehealth application and the design of our evaluating study. For example, improvements have already been made to the ePROM delivery system to ensure that all participants are able to receive and complete these validated questionnaires with increased ease and accessibility.

Several limitations are present in this study and should be taken into consideration. As a proof-of-concept study (IDEAL stage 1), we applied a small sample size, and therefore, lacked a comparator group. As set out by the IDEAL framework, stages 2 and 3 studies would adopt a larger sample size before trying to formally assess outcomes. Ideally, all patients would have been onboarded prior to their surgical procedure but due to adjustments made in the admission process during the COVID-19 pandemic, this was not always possible. Given two of the six participants withdrew and the pragmatic nature of recruitment, further studies should have a more robust recruitment approach for repeatability, but also capture more information (where possible and in a non-obtrusive manner) around reasons for withdrawal. Generally, a lack of ePRO data is available in this study due to retention rate and technical issues with the deployment tool; however, this should be managed in the next iteration of the study with a larger cohort, updated application (bug fix) and longer follow-up. Going forward, a more inclusive approach should be taken to include the multidisciplinary team. This could be achieved with the newly developed HCP dashboard to enable real time data review, as well as the collection and evaluation of HCP feedback.

## Conclusion

This study provides a pivotal first step in the evidence-based evaluation of a mobile health application for the holistic support of patients living with brain cancer. Our results indicate that a larger study is feasible, safe, but also, necessary, to determine the clinical impact such a tool may have. Initial results indicate that patients using the Vinehealth application feel a subjective improvement in their care and are optimistic about its use. The findings from this study can inform the design of larger studies to assess the impact of mHealth tools more rigorously and objectively.

## Data Availability

All data relevant to the study are included in the article or uploaded as online supplemental information.
